# Induction of Apoptotic Cell Death by Oral Streptococci in Human Periodontal Ligament Cells

**DOI:** 10.3389/fmicb.2021.738047

**Published:** 2021-10-14

**Authors:** Ok-Jin Park, A Reum Kim, Yoon Ju So, Jintaek Im, Hyun Jung Ji, Ki Bum Ahn, Ho Seong Seo, Cheol-Heui Yun, Seung Hyun Han

**Affiliations:** ^1^Department of Oral Microbiology and Immunology and Dental Research Institute, School of Dentistry, Seoul National University, Seoul, South Korea; ^2^Research Division for Radiation Science, Korea Atomic Energy Research Institute, Jeongeup, South Korea; ^3^Department of Agricultural Biotechnology, Research Institute of Agriculture and Life Sciences, Seoul National University, Seoul, South Korea; ^4^Institute of Green Bio Science and Technology, Seoul National University, Pyeongchang, South Korea

**Keywords:** apoptosis, streptococcal species, human periodontal ligament cells, hydrogen peroxide, lipoproteins, reactive oxygen species

## Abstract

Initiation and progression of oral infectious diseases are associated with streptococcal species. Bacterial infection induces inflammatory responses together with reactive oxygen species (ROS), often causing cell death and tissue damage in the host. In the present study, we investigated the effects of oral streptococci on cytotoxicity and ROS production in human periodontal ligament (PDL) cells. *Streptococcus gordonii* showed cell cytotoxicity in a dose- and time-dependent manner. The cytotoxicity might be due to apoptosis since *S. gordonii* increased annexin V-positive cells, and the cytotoxicity was reduced by an apoptosis inhibitor, Z-VAD-FMK. Other oral streptococci such as *Streptococcus mitis*, *Streptococcus sanguinis*, and *Streptococcus sobrinus* also induced apoptosis, whereas *Streptococcus mutans* did not. All streptococci tested except *S. mutans* triggered ROS production in human PDL cells. Interestingly, however, streptococci-induced apoptosis appears to be ROS-independent, as the cell death induced by *S. gordonii* was not recovered by the ROS inhibitor, resveratrol or *n*-acetylcysteine. Instead, hydrogen peroxide (H_2_O_2_) appears to be important for the cytotoxic effects of streptococci since most oral streptococci except *S. mutans* generated H_2_O_2_, and the cytotoxicity was dramatically reduced by catalase. Furthermore, streptococcal lipoproteins are involved in cytotoxicity, as we observed that cytotoxicity induced by the lipoprotein-deficient *S. gordonii* mutant was less potent than that by the wild-type and was attenuated by anti-TLR2-neutralizing antibody. Indeed, lipoproteins purified from *S. gordonii* alone were sufficient to induce cytotoxicity. Notably, *S. gordonii* lipoproteins did not induce H_2_O_2_ or ROS but cooperatively induced cell death when co-treated with H_2_O_2_. Taken together, these results suggest that most oral streptococci except *S. mutans* efficiently induce damage to human PDL cells by inducing apoptotic cell death with bacterial H_2_O_2_ and lipoproteins, which might contribute to the progression of oral infectious diseases such as apical periodontitis.

## Introduction

Streptococci are commensal Gram-positive aerobic bacteria found in human body such as skin, oral cavity, and intestine (Abranches et al., 2018). At the same time, they can act as opportunistic pathogens causing infectious diseases such as apical periodontitis, pneumonia, sepsis, and skin infections (Parks et al., 2015). Among them, oral streptococci including *S. gordonii*, *S. mutans*, *S. mitis*, and *S. sanguinis* are commonly found in the human oral cavity (Abranches et al., 2018). They have been demonstrated to cause systemic diseases such as bacteremia, sepsis, and infective endocarditis (Park et al., 2020). Oral streptococci, as early colonizers of the oral cavity, have been isolated from infected root canals of patients with apical periodontitis (Chavez de Paz et al., 2005) and are known to cause inflammation and tissue destruction in periapical lesions (Kutlu et al., 2003). Metagenomic analysis showed that *Streptococcus* is a predominant genus in patients with gingivitis (Park et al., 2015). Oral streptococci have been shown to induce macrophage cell death through hydrogen peroxide (H_2_O_2_) production (Okahashi et al., 2013). We previously reported that *S. gordonii* efficiently produces nitric oxide and proinflammatory cytokines in macrophages and induces bone destruction by stimulating osteoclastogenesis while inhibiting osteoblastogenesis (Kim et al., 2017b, 2018; Park et al., 2019).

Bacterial infections in the oral cavity induce inflammatory responses that often cause destruction of tissues such as the periodontal ligament (PDL), pulp, and alveolar bone (Cekici et al., 2014). The PDL, a type of connective tissue between teeth and alveolar bone, includes fibroblasts (the most predominant cells), epithelial cells, and osteoblasts (Jonsson et al., 2011). It is well known that PDL cells interact with bacteria in the periodontal pocket and periapical lesions and are associated with inflammatory responses (Cekici et al., 2014). For example, periodontopathic bacteria such as *Porphyromonas gingivalis* have been reported to induce inflammatory cytokines including IL-1β, IL-8, and TNF-α in PDL cells (Yamamoto et al., 2006). We also previously reported increase in IL-8 expression in PDL cells treated with *S. gordonii* (Kim et al., 2017a) or *Aggregatibacter actinomycetemcomitans* lipopolysaccharide (LPS) (Im et al., 2015). In addition, PDL cells treated with *P. gingivalis* LPS exhibit increased production of reactive oxygen species (ROS) (Golz et al., 2014). Furthermore, it has been reported that the upregulation of receptor activator of NF-kappa B ligand (RANKL) by *A. actinomycetemcomitans* LPS in PDL cells contributes to the pathogenesis of periodontitis (Tiranathanagul et al., 2004). Therefore, effector molecules produced during the interactions between pathogenic bacteria and PDL cells seem to be important for the development of periodontitis.

ROS are induced by reduction of molecular oxygen in the mitochondria under normal physiological conditions (Circu and Aw, 2010). ROS include free radicals such as superoxide anion and hydroxyl radical and non-radicals such as H_2_O_2_ and singlet oxygen (Li et al., 2016). ROS contribute to cell proliferation, differentiation, and inflammation through regulation of intracellular signaling (Circu and Aw, 2010). Moderate ROS production induces inflammatory responses for host defense (Wang et al., 2014). In contrast, excessive ROS production by the inflammatory lesion can damage nucleic acids, proteins, and lipids and eventually lead to tissue injury *via* cellular damage and apoptosis (Circu and Aw, 2010; Mittal et al., 2014). ROS-independent apoptosis has been also reported (Seong and Lee, 2018). It has been suggested that hyper-production of ROS is associated with pathologies in various diseases including cancer, atherosclerosis, and diabetes (Brieger et al., 2012; Kehrer and Klotz, 2015). Periodontitis often induces excessive ROS in periodontal tissues (Akalin et al., 2007), but the underlying molecular mechanism is not clear. Therefore, in this study, we investigated the effects of various oral streptococci on cytotoxicity and ROS production in PDL cells.

## Materials and Methods

### Reagents and Chemicals

*S. gordonii* CH1 and *S. mitis* SF100 were used as previously described (Seo et al., 2010; Kim et al., 2017a). *S. mutans* KCTC3065 and *S. sanguinis* KCTC3284 were obtained from the Korean Collection for Type Cultures (Jeongeup, Korea). *S. sobrinus* NIDR 6715-7 was provided by Prof. Bong-Kyu Choi (Seoul National University, Seoul, Korea). Lipoprotein-deficient (*Δlgt*) and lipoteichoic acid (LTA)-deficient (*ΔltaS*) *S. gordonii* and lipoproteins were prepared from *S. gordonii* CH1 as previously described (Kim et al., 2017b). Todd–Hewitt broth, brain heart infusion (BHI), and yeast extract were purchased from BD Biosciences (San Diego, CA, United States). Minimum Essential Medium, alpha modification (α-MEM), Dulbecco’s Modified Eagle’s Medium (DMEM), and phosphate-buffered saline (PBS) were obtained from WelGENE (Daegu, Korea). Penicillin/streptomycin was purchased from HyClone (Logan, UT, United States). Fetal bovine serum (FBS), trypsin–EDTA, and trypan blue were purchased from Gibco-BRL (Carlsbad, CA, United States). 2',7'-Dichlorofluorescin diacetate (DCF-DA), resveratrol, *n*-acetylcysteine (NAC), and catalase were purchased from Sigma-Aldrich (St. Louis, MO, United States). Z-VAD-FMK was purchased from InvivoGen (San Diego, CA, United States). Dead cell apoptosis kit was purchased from Invitrogen (Carlsbad, CA, United States).

### Bacteria Culture

Wild-type *S. gordonii* CH1, lipoprotein-deficient (*Δlgt*) *S. gordonii*, LTA-deficient (*ΔltaS*) *S. gordonii* in Todd-Hewitt broth with 5% yeast extract (THY), and *S. mutans*, *S. mitis*, *S. sobrinus*, and *S. sanguinis* in BHI were grown at 37°C under static condition. The bacteria were diluted 1:100 in fresh medium, cultured to mid-log phase, and then washed with PBS. The cells were grown in α-MEM containing 10% FBS and 1% penicillin/streptomycin at 37°C in a 5% CO_2_ incubator.

### Isolation and Culture of PDL Cells

All experiments using healthy human PDL cells were approved by the Institutional Review Board at Seoul National University. The wisdom teeth were obtained from young adults (two males and one female, average 23years old) at the Dental Hospital of Seoul National University, Korea. PDL tissue was removed from the root surface of teeth and chopped into the small pieces. The tissue was digested with 0.25% trypsin and 1mM EDTA in PBS for 1h at 37°C and vortexed every 10min. To obtain the single cells, the digested tissue suspension was passed through a 70-μm cell strainer (BD Falcon, Franklin Lakes, NJ, United States). The cells were collected by centrifugation and cultured in DMEM containing 10% heat-inactivated FBS, 100U/ml penicillin, and 100μg/ml streptomycin at 37°C in a 5% CO_2_ humidified incubator. The PDL cells were treated with streptococci at various multiplicities of infection (MOI) for 1, 3, or 6h in the complete culture media without antibiotics. Primary human PDL cells at passages four to nine were used in this study.

### Cell Viability Test

Human PDL cells (3×10^5^ cells/ml, 2ml) were plated onto 60-mm dishes and stimulated with *S. gordonii* at MOI 1:10, 100, or 1,000 for 1, 3, or 6h. In separate experiments, the cells were pre-treated with Z-VAD-FMK or resveratrol for 1h and then stimulated with *S. gordonii* for 3h. The cells were washed with PBS, detached using trypsin–EDTA, and stained with trypan blue. Live (unstained) and dead (stained) cells were enumerated under light microscopy. The cells were counted within 5min after the mixing with trypan blue.

### Cell Apoptosis Assay

Cell death staining was conducted according to the manufacturer’s instructions (Invitrogen). Briefly, human PDL cells (3×10^5^ cells/ml, 2ml) were plated onto 60-mm dishes and stimulated with *S. gordonii* at MOI of 1,000 for 30, 60, or 180min. Then, the cells were treated with EDTA for 5min, detached with a scraper, and harvested. After washing twice with PBS, the cells were stained with FITC-annexin V and propidium iodide (PI) in the dark for 15min. The staining of cells was analyzed by flow cytometer (BD Biosciences) using Flow Jo software (Tree Star, San Carlos, CA).

### Intracellular ROS Detection

Intracellular ROS were detected using DCF-DA as described previously (Song et al., 2021). Briefly, human PDL cells (3×10^5^ cells/ml, 2ml) were plated onto 60-mm dishes. The cells were detached with trypsin–EDTA, washed with PBS, and treated with 10μM of DCF-DA for 30min at 37°C. DCF-DA-treated cells were washed twice with PBS, followed by stimulation with *S. gordonii* at MOI of 1:10, 100, or 1,000 for 3h. After washing, the ROS levels were analyzed using a flow cytometer (BD Biosciences).

### H_2_O_2_ Measurement

Human PDL cells (3×10^5^ cells/ml, 2ml) were plated onto 60-mm dishes and treated with oral streptococci for 1h. The supernatants were harvested by centrifugation at 10,000×*g* for 5min. Level of H_2_O_2_ in the supernatant was assayed according to the manufacturer’s instructions (EZ-hydrogen peroxide/peroxidase assay kit, DoGenBio, Seoul, Korea). H_2_O_2_ was quantified by measuring the absorbance at 560nm using a microplate reader (Molecular Devices, San Jose, CA, United States).

### Statistical Analysis

All experiments were performed three to five times. All data are expressed as mean±standard deviation (SD) of triplicate samples. Statistical significance was examined with a nonparametric Mann–Whitney test. An asterisk (^*^) indicates a significant difference, defined as *p*<0.05.

## Results

### *S. gordonii* Exhibits Cytotoxicity to Human Periodontal Ligament Cells

**Figure 1:**
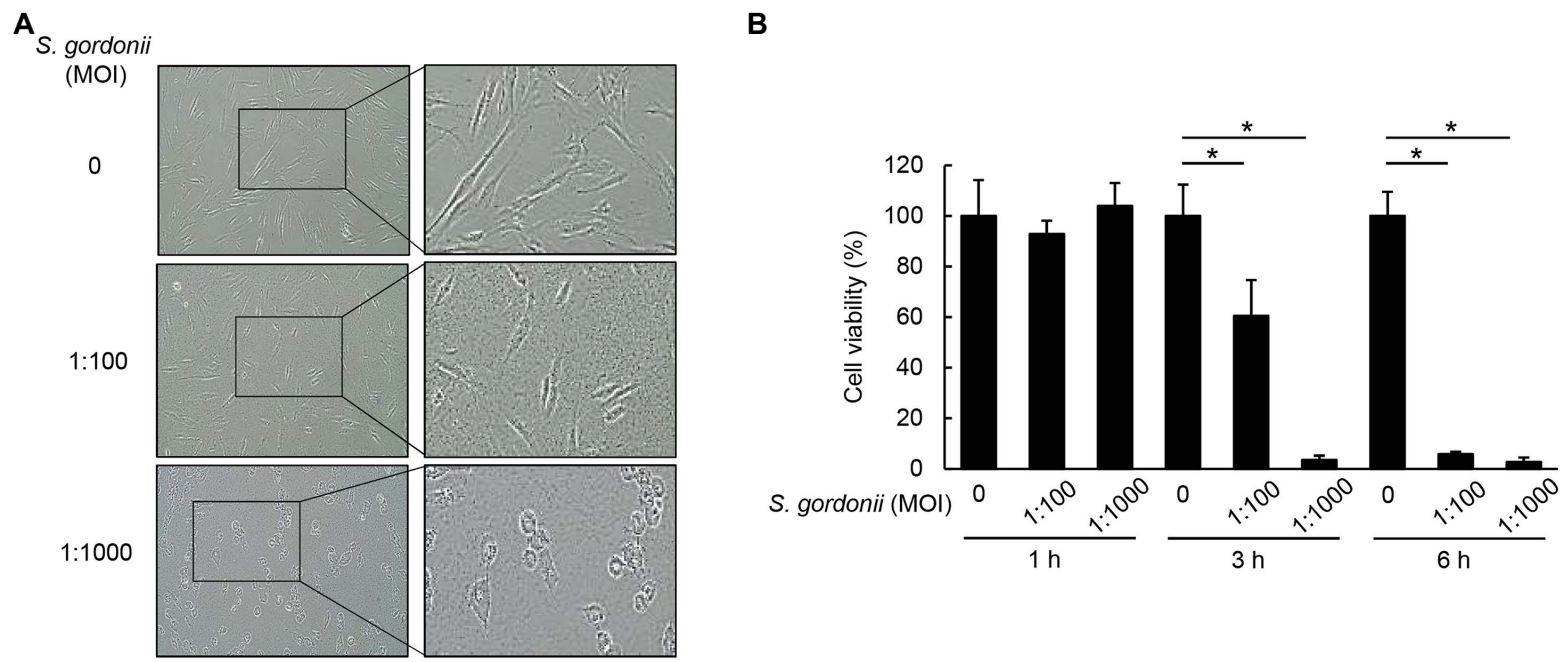
*S. gordonii* induces cytotoxicity of periodontal ligament (PDL) cells. *S. gordonii* was cultured to mid-log phase in THY media at 37°C. PDL cells were treated with *S. gordonii* at multiplicities of infection (MOI) 1:10, 1:100, or 1:1,000 for 1, 3, or 6h. Trypan blue assay was used to determine the number of viable PDL cells. The cells were photographed **(A)** and counted **(B)**. One of three similar results is shown. ^*^p<0.05.

We examined the effect of *S. gordonii* on the viability of human PDL cells. The cells treated with live *S. gordonii* exhibited morphological changes typical of dying cells and were detached from the culture dish (Figure 1A). Cytotoxicity was dose- and time-dependent (Figure 1B). In addition, *S. gordonii* efficiently induced cell death in primary PDL cells taken from different donors (Supplementary Figure 1). These results suggest that *S. gordonii* induces damage in human PDL cells to result in cell death.

### *S. gordonii* Induces Apoptotic Cell Death Through ROS-Independent Pathways

**Figure 2:**
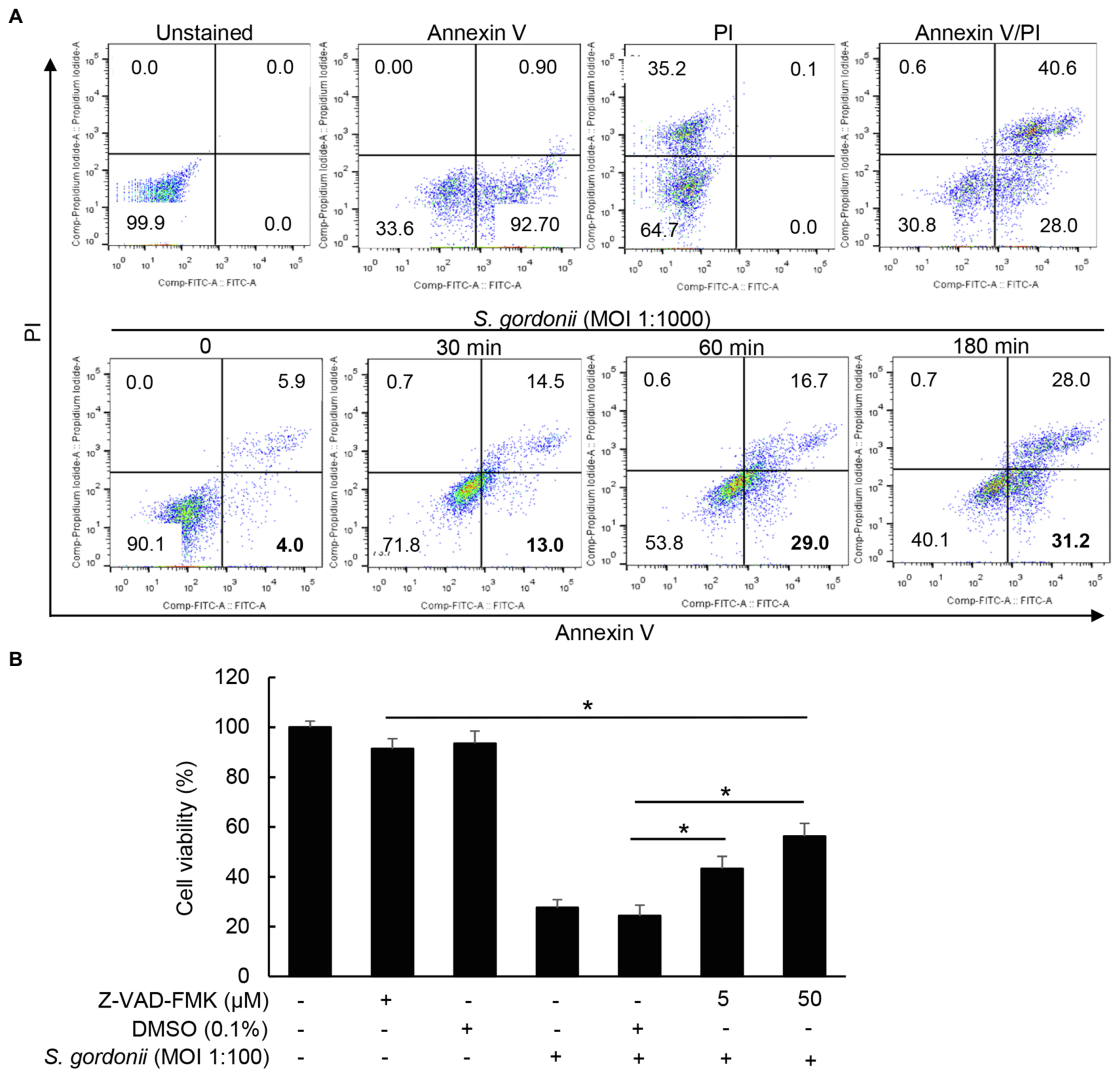
Cytotoxicity by *S. gordonii* is mediated through apoptosis in PDL cells. *S. gordonii* was cultured to mid-log phase in THY media at 37°C. **(A)** PDL cells were treated with *S. gordonii* at MOI 1:1,000 for 30, 60, or 180min. The cells were stained with annexin V and PI and then analyzed using flow cytometry. Numbers indicate the percentage of cells in each panel. **(B)** PDL cells were pre-treated with Z-VAD-FMK (0, 5, or 50μM) for 1h, followed by treatment with *S. gordonii* at MOI 1:100 for 3h. Trypan blue assay was used to determine the number of viable PDL cells. The cells were counted. One of three similar results is shown. ^*^p<0.05.

**Figure 3:**
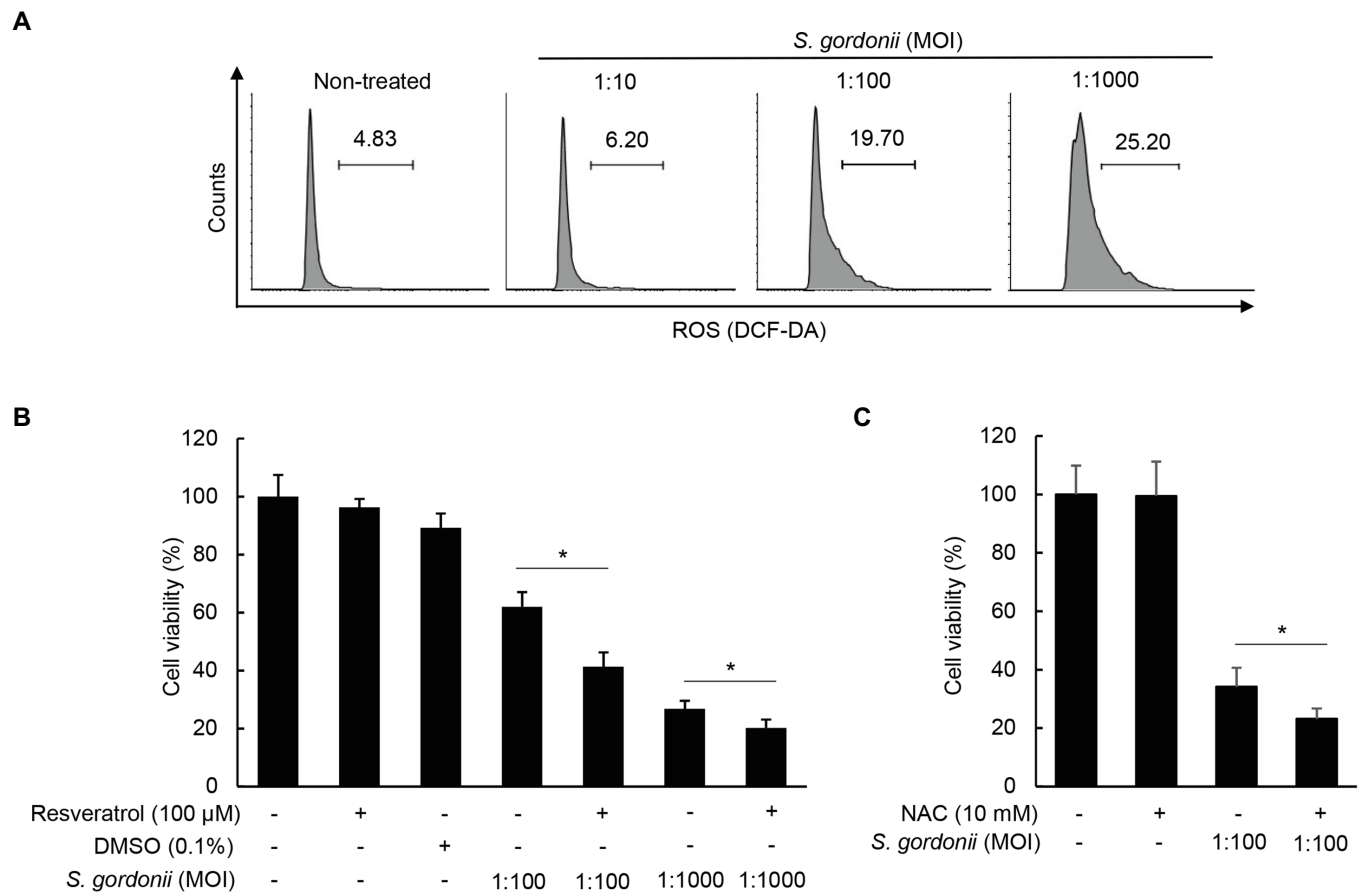
*S. gordonii* induces reactive oxygen species (ROS) production regardless of its cytotoxic effects in PDL cells. **(A)** PDL cells were treated with 10μM of DCF-DA for 30min at 37°C. After washing with PBS, the DCF-DA-labeled cells were treated with *S. gordonii* at MOI 1:10, 1:100, or 1:1,000 for 3h. Fluorescent intensity was analyzed by flow cytometry. **(B)** PDL cells were pre-treated with 100μM of resveratrol for 1h, followed by treatment with *S. gordonii* at MOI 1:100 or 1:1,000 for 3h. **(C)** PDL cells were pre-treated with 10mM NAC for 1h, followed by treatment with *S. gordonii* at MOI 1:100 for 3h. Trypan blue assay was used to determine the number of viable cells. One of three similar results is shown. ^*^p<0.05.

Next, annexin V/PI staining was performed to determine whether cell death induced by *S. gordonii* was due to apoptosis or necrosis. As shown in Figure 2A, *S. gordonii* increased annexin V-positive cells (apoptotic cells) and annexin V/PI double-positive cells (both late apoptotic and necrotic cells) but decreased annexin V/PI double-negative cells (viable cells) in a time-dependent manner. However, when the cells were pre-treated with Z-VAD-FMK, an apoptosis inhibitor, *S. gordonii* did not inhibit cell viability (Figure 2B). These results indicate that *S. gordonii*-induced PDL cell death is mediated through apoptosis. On the other hand, excessive ROS production can lead to cell apoptosis (Circu and Aw, 2010) Thus, we hypothesized that *S. gordonii* induces ROS-mediated apoptosis in PDL cells. When the cells were treated with *S. gordonii*, ROS production of PDL cells was increased in a dose-dependent manner (Figure 3A). However, ROS inhibitor resveratrol or *n*-acetylcysteine did not affect cytotoxicity of PDL cells by *S. gordonii* (Figures 3B,C). These results suggest that *S. gordonii* induces ROS generation but is not related to PDL cell death.

### Other Streptococcal Species Except *S. mutans* Also Induce Apoptosis and ROS Production of Human PDL Cells

**Figure 4:**
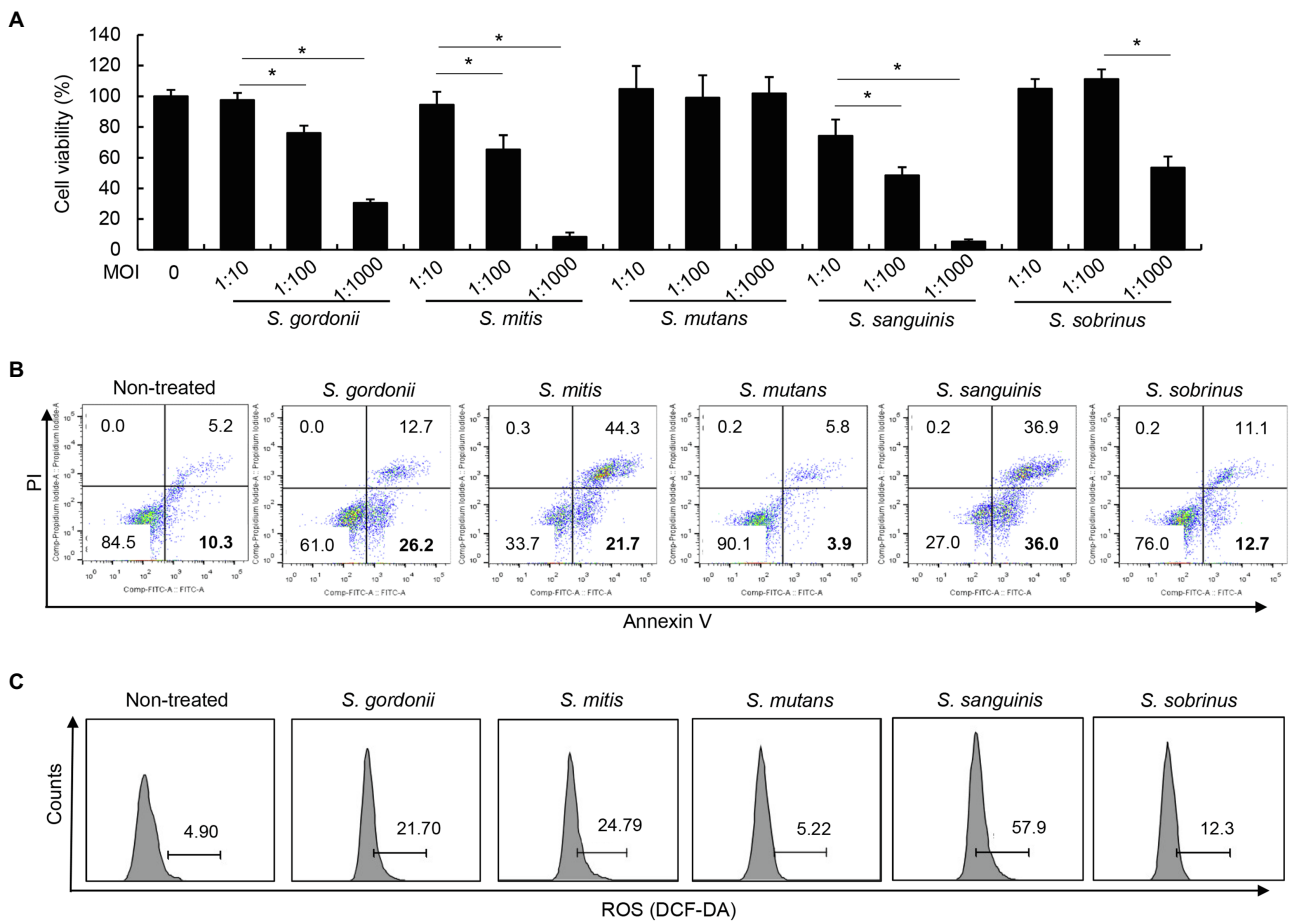
Various streptococcal species differ in induction of apoptosis and ROS in human PDL cells. **(A)** PDL cells were treated with *S. gordonii*, *S. mitis*, *S. mutans*, *S. sanguinis*, and *S. sobrinus* at MOI 1:10, 1:100, or 1:1,000 for 3h. Trypan blue assay was used to determine the number of viable cells. **(B)** PDL cells were treated with *S. gordonii*, *S. mitis*, *S. mutans*, *S. sanguinis*, and *S. sobrinus* at MOI 1:1,000 for 1h. The cells were strained with annexin V and PI and then analyzed using flow cytometry. **(C)** PDL cells were treated with 10μM of DCF-DA for 30min at 37°C. The DCF-DA-treated cells were washed with PBS and then treated with *S. gordonii*, *S. mitis*, *S. mutans*, *S. sanguinis*, and *S. sobrinus* at MOI 1:1,000 for 3h in a CO_2_ incubator. Fluorescent intensity was analyzed by flow cytometry. One of three similar results is shown. ^*^p<0.05.

To examine whether other streptococci can also induce apoptosis, human PDL cells were treated with *S. gordonii*, *S. mitis*, *S. mutans*, *S. sanguinis*, and *S. sobrinus* for 3h. Interestingly, *S. gordonii*, *S. mitis*, *S. sanguinis*, and *S. sobrinus* induced PDL cell cytotoxicity (Figure 4A) and annexin V-positive cell population (Figure 4B). However, such effects were not observed in PDL cells treated with *S. mutans* under the same conditions (Figures 4A,B). Concordant with their cytotoxic effect on human PDL cells, *S. gordonii*, *S. mitis*, *S. sanguinis*, and *S. sobrinus* but not *S. mutans* induced ROS production (Figure 4C). These results suggest that most streptococcal species, except *S. mutans*, induce apoptotic cell death and ROS generation in human PDL cells.

### Streptococci-Induced PDL Cell Cytotoxicity Is Mediated Through H_2_O_2_ Production

**Figure 5:**
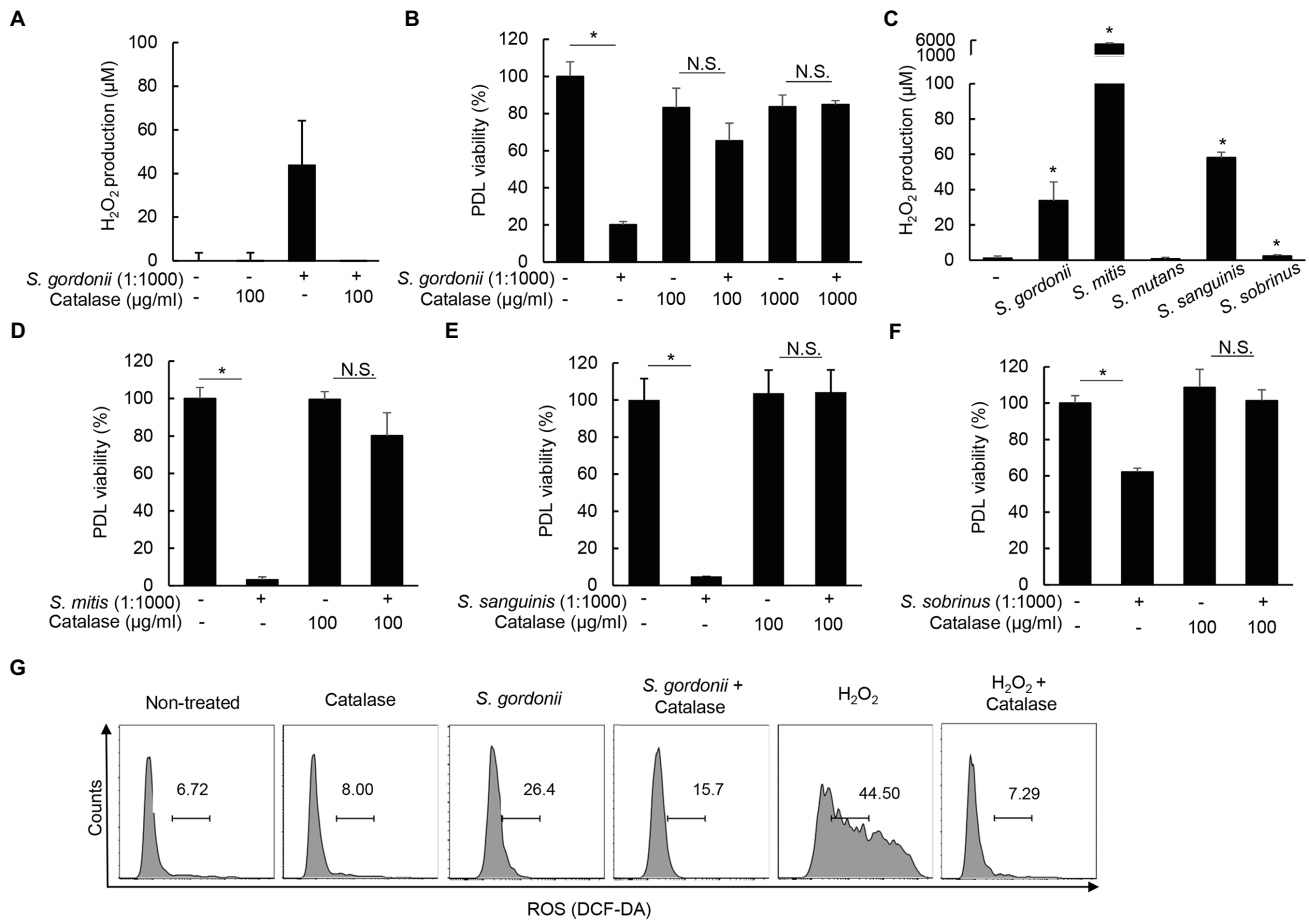
*Streptococci*-induced cytotoxicity of PDL cells is mediated through production of H_2_O_2_. **(A)** PDL cells were treated with *S. gordonii* at MOI 1:1,000 in the presence or absence of catalase (100μg/ml) for 1h. The level of H_2_O_2_ in the supernatant was determined using a hydrogen peroxide assay kit. **(B)** PDL cells were treated with *S. gordonii* at MOI 1:1,000 in the presence or absence of catalase (100 or 1,000μg/ml) for 3h. Trypan blue assay was used to determine the number of viable cells. **(C)** PDL cells were treated with *S. gordonii, S. mitis, S. mutans, S. sanguinis*, or *S. sobrinus* at MOI 1:1,000 for 1h. The level of H_2_O_2_ in the supernatant was determined using a hydrogen peroxide assay kit. **(D–F)** PDL cells were treated with *S. mitis, S. sanguinis*, or *S. sobrinus* in the presence or absence of catalase for 3h. Trypan blue assay was used to determine the number of viable cells. **(G)** PDL cells were treated with 10μM of DCF-DA for 30min at 37°C. DCF-DA-treated cells were washed with PBS and then treated with *S. gordonii* at MOI 1:1,000 or H_2_O_2_ in the presence or absence of catalase for 3h. Fluorescent intensity was analyzed by flow cytometry. One of three similar results is shown. ^*^p < 0.05.

Oral streptococci have been reported to induce cell death of macrophages *via* production of H_2_O_2_ (Okahashi et al., 2013). Thus, we examined whether the reduction of PDL cell viability was due to the cytotoxicity of streptococci-produced H_2_O_2_. As shown in Figure 5A, *S. gordonii* induced H_2_O_2_, which was completely inhibited by catalase treatment. When human PDL cells were treated with *S. gordonii* in the presence of catalase, *S. gordonii*-induced PDL cytotoxicity was reversed substantially (Figure 5B). Likewise, *S. mitis*, *S. sanguinis*, and *S. sobrinus* also produced H_2_O_2_, but *S. mutans* did not (Figure 5C). The reduction of cell viability by H_2_O_2_-producing streptococci was recovered by catalase treatment (Figures 5D–F), suggesting that oral streptococci induce the death of human PDL cells *via* H_2_O_2_ production. In addition, when human PDL cells were co-treated with *S. gordonii* and catalase, ROS was still produced in human PDL cells (Figure 5G). These results suggest that *S. gordonii*-produced H_2_O_2_ does not significantly affect the generation of ROS in PDL cells.

### *Streptococcus gordonii* Lipoproteins Are Partially Involved in the Cytotoxicity of Human PDL Cells

**Figure 6:**
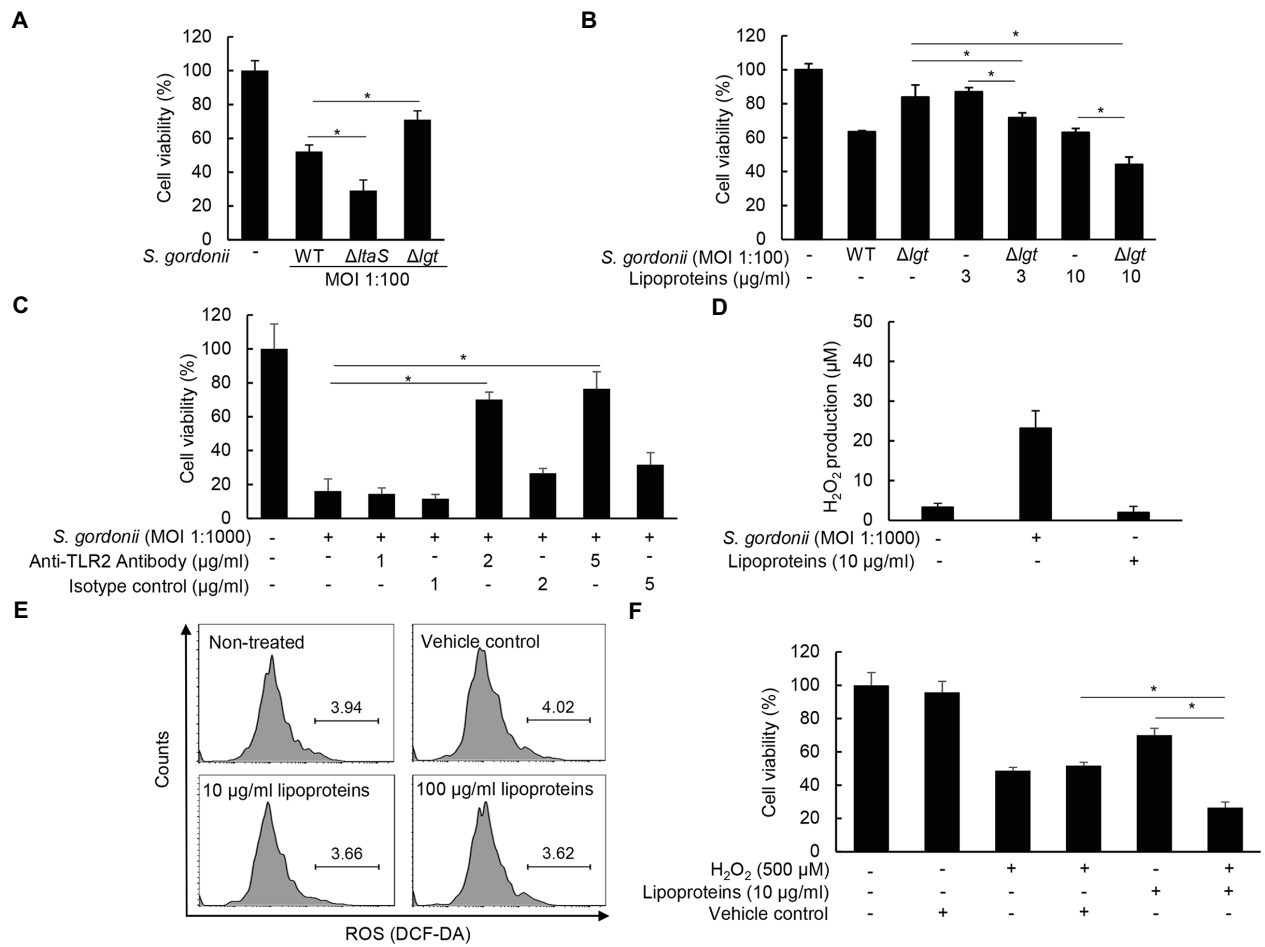
*S. gordonii* lipoproteins contribute to the cytotoxicity of PDL cells in cooperation with H_2_O_2_. **(A)** PDL cells were treated with *S. gordonii* wild-type, its *ΔltaS*, and *Δlgt* at MOI 1:100 for 3 h. **(B)** PDL cells were treated with *S. gordonii* wild-type or its *Δlgt* at MOI 1:100 in the presence or absence of *S. gordonii* lipoproteins (3 or 10 μg/ml) for 3 h. **(C)** PDL cells were pre-treated with anti-TLR2 antibody (0, 1, 2, or 5 μg/ml) for 1 h, followed by treatment with *S. gordonii* at MOI 1:1000 for 3 h. Trypan blue assay was used to determine the number of viable cells. **(D)** PDL cells were treated with *S. gordonii* at MOI 1:1000 or S. gordonii lipoproteins (10 μg/ml) for 1 h. The level of H_2_O_2_ in the supernatant was determined using a hydrogen peroxide assay kit. **(E)** PDL cells were treated with 10 µM of DCF-DA for 30 min at 37°C. The DCF-DA-treated cells were washed with PBS and then treated with *S. gordonii* lipoproteins (10 or 100 μg/ml) for 3 h in a CO_2_ incubator. Fluorescent intensity was analyzed by flow cytometry. **(F)** PDL cells were treated with *S. gordonii* lipoproteins (10μg/ml) in the presence or absence of H_2_O_2_ for 3h. Trypan blue assay was used to determine the number of viable cells. One of three similar results is shown. ^*^p < 0.05.

Lipoproteins are a major virulence factor of Gram-positive bacteria (Kim et al., 2018), and over-activation of toll-like receptor 2 (TLR2) by sensing bacterial lipoproteins often results in cytotoxicity (Aliprantis et al., 2000). Indeed, bacterial lipoproteins have been shown to induce cell death *via* TLR2 in THP-1 cells (Aliprantis et al., 1999). Thus, we examined whether bacterial lipoproteins also contribute to streptococci-induced PDL cell cytotoxicity with *Δlgt*. As shown in Figure 6A, *Δlgt S. gordonii* showed decreased cytotoxicity in PDL cells compared to wild-type *S. gordonii*. In contrast, *ΔltaS S. gordonii*-treated cells showed even higher cytotoxicity than wild-type *S. gordonii*. In addition, treatment with lipoproteins purified from *S. gordonii* also decreased the viability of PDL cells. When cells were co-treated with *Δlgt S. gordonii* and lipoproteins purified from *S. gordonii*, the extent of cytotoxicity was comparable to that induced by wild-type *S. gordonii* (Figure 6B). Moreover, when PDL cells were pre-treated with anti-human TLR2 neutralizing antibody, *S. gordonii*-decreased cell viability was recovered substantially (Figure 6C). However, such recovery was not seen in the presence of isotype control antibody. These results suggest that TLR2 is a critical factor for inhibition of cell viability by *S. gordonii*. On the other hand, *S. gordonii* lipoproteins are unable to induce H_2_O_2_ (Figure 6D) or ROS (Figure 6E) generation, indicating that lipoprotein-induced cytotoxicity is independent of H_2_O_2_ or ROS. In addition, *S. gordonii* lipoproteins and H_2_O_2_ cooperatively induced PDL cell cytotoxicity (Figure 6F). Collectively, these results suggest that *S. gordonii* lipoproteins contribute to the cytotoxicity of PDL cells in cooperation with H_2_O_2_.

## Discussion

Apical periodontitis is characterized by inflammation and tissue injury in the lesion (Nair, 2004). Since PDL cells play critical roles in the support of teeth in the alveolar bone (Jonsson et al., 2011), damage to PDL cells might contribute to the pathogenesis of apical periodontitis. *S. gordonii* is a medically important bacterium that enters the bloodstream through oral cavity and causes systemic diseases (Park et al., 2020). In this study, we demonstrated that most oral streptococcal species including *S. gordonii* could damage PDL cells by inducing apoptotic cell death. Mechanism studies suggest that streptococcal H_2_O_2_ generation is crucial for PDL cytotoxicity, while cell death was independent of ROS production. In addition, streptococcal lipoproteins also contribute to cytotoxic effects on PDL cells. Considering that streptococcal species are found predominantly in the initial stage of periodontal damage (Park et al., 2015) and refractory apical periodontitis (Chavez de Paz et al., 2005), the current results demonstrate that *S. gordonii* plays an important role in the early stage of inflammation by inducing cytotoxicity in PDL cells.

We found that *S. gordonii* induces apoptotic cell death of human PDL cells, as demonstrated by pre-treatment with apoptosis inhibitor attenuating *S. gordonii*-induced apoptosis. In fact, bacteria-induced host cell damage *via* apoptosis is not uncommon in apical periodontitis. For example, *Enterococcus faecalis*, which is associated with refractory apical periodontitis (Wang et al., 2012a), has been shown to induce apoptosis and pyroptosis in human osteoblastic MG63 cells (Ran et al., 2019), suggesting that *E. faecalis* infection in periapical lesions contributes to delay in periapical repair. In addition, diabetic rats treated with oral administration of *A. actinomycetemcomitans* exhibit increased numbers of apoptotic cells in the PDL adjacent to the bone and lining on the bone surface. However, when *A. actinomycetemcomitans*-inoculated diabetic rats receive the apoptosis inhibitor ZDEVD-FMK, bone destruction is decreased *via* increased osteoblast numbers in PDL (Pacios et al., 2013). Since PDL cells play a critical role in tooth attachment to the surrounding alveolar bone (Jonsson et al., 2011), apoptosis of PDL cells is likely to contribute to tooth loss *via* apical periodontitis.

We observed that H_2_O_2_ production by various streptococci is critical for cell death of human PDL cells. In this study, when the cells were co-treated with catalase and H_2_O_2_-producing streptococci, the reduction of cell viability by streptococci was recovered almost completely. As in the present study, oral streptococci have been shown to induce macrophage cell death through H_2_O_2_ generation (Okahashi et al., 2013), and streptococci-induced H_2_O_2_ is independent of inflammatory responses. It is well known that exogenous H_2_O_2_ induces apoptosis of various host cells including rat primary neuronal cell culture and human pulmonary artery smooth muscle cells (Whittemore et al., 1995; Park, 2016). Rai et al. reported that H_2_O_2_ produced by *Streptococcus pneumoniae* contributes to acute pneumonia *via* DNA damage and apoptosis of lung cells (Rai et al., 2015). Therefore, the cytotoxicity of H_2_O_2_ by oral streptococci can contribute to the pathogenicity in infectious diseases *via* tissue damage. In addition, because streptococcus pyruvate oxidase (spxB) is critical for the induction of H_2_O_2_ by oral streptococci (Zhu et al., 2014), further studies using spxB mutant *S. gordonii* are needed to clearly understand the role of H_2_O_2_ produced by *S. gordonii* on cell death.

In our study, *S. gordonii*, like many other bacteria previously reported, increased ROS production in PDL cells. For example, under physiological conditions, commensal gut microbiota induce ROS in intestinal epithelial cells (Jones et al., 2012). *E. faecalis* increases ROS production in gastric carcinoma cell line MKN74 (Strickertsson et al., 2013). *P. gingivalis* and *A. actinomycetemcomitans* induce ROS in gingival epithelial cells and macrophages, respectively (Wang et al., 2014; Okinaga et al., 2015). However, under excessive stress, host cells produce high amounts of ROS, often leading to cell death (He et al., 2017). ROS induction by *Streptococcus oralis* and *Staphylococcus aureus* has been shown to cause host cell death (Okahashi et al., 2013; Deplanche et al., 2019). We found that *S. gordonii*-induced ROS production is not associated with apoptosis of PDL cells. Thus, further studies are needed to determine the ROS requirements for apoptosis by bacterium. On the other hand, Marconi et al. reported the protective effect of ascorbic acid in primary culture of human periodontal ligament stem cells (PDLSCs) exposed to *P. gingivalis* LPS through down-regulation of inflammatory pathway and ROS induction (Marconi et al., 2021). It would be necessary to study streptococci-induced cytotoxicity in other cell types including PDLSCs and human dental pulp stem cells.

In the present study, we demonstrated that all streptococci do not affect the viability of PDL cells to similar extents. *S. gordonii*, *S. mitis*, *S. sanguinis*, and *S. sobrinus* but not *S. mutans* induced apoptosis of PDL cells. In general, it is well known that *S. mutans* induces dental caries (Forssten et al., 2010). Since *S. mutans* is an early colonizer that converts sucrose into sticky glucan and causes other bacteria to adhere to it (Ren et al., 2016), its action seems to be largely limited to hard tissues such as teeth. Instead, accompanying bacteria that arrive after *S. mutans* can more efficiently enter the pulp and consequently cause inflammation by inducing cell cytotoxicity. Nevertheless, *S. mutans* LTA has been shown to induce apoptosis in human dental pulp cells (Wang et al., 2001). Since LTA can be released by Gram-positive bacteria (Ginsburg, 2002), bacterial components derived from streptococcal species including *S. mutans* can contribute to host cell damage or inflammatory responses, although the bacteria do not directly contribute. On the other hand, cell tropism in differential responses to bacteria might be another reason for the variation in results. For example, *S. gordonii* does not induce apoptosis in gingival keratinocytes (Li et al., 2013) even though it induced apoptotic cell death in human PDL cells in the current study. Although further studies are required to clarify our observations, most oral streptococci might contribute to apical periodontitis through death of PDL cells.

We demonstrated that lipoproteins are involved at least partially in PDL cell cytotoxicity by *S. gordonii via* TLR2 activation, although lipoproteins have weak cytotoxic effects compared to H_2_O_2_. The strong TLR2 signaling caused by high doses of lipoproteins might be cytotoxic to a level similar to that of H_2_O_2_. TLR2 activation seems to be a positive modulator of apoptosis. Aliprantis et al. reported that a synthetic lipopeptide, Pam3CSK4, mimicking bacterial lipoproteins induces apoptosis of THP-1 cells through activation of TLR2/MyD88-NF-κB and a Fas-associated death domain protein/caspase 8 pathway (Aliprantis et al., 1999, 2000). Additionally, *Propionibacterium acnes* has been shown to induce the apoptosis of nucleus pulposus cells isolated from human intervertebral discs by the TLR2/c-Jun *N*-terminal kinase pathway (Lin et al., 2018). Indeed, lipoproteins of streptococci are important inflammatory components, based on the previous studies in which we found that lipoprotein-deficient *S. gordonii* weakly stimulates the induction of inflammatory mediators compared with the wild-type strain, and purified lipoproteins are sufficient to induce inflammatory responses in macrophages and PDL cells (Kim et al., 2017a, 2018). Therefore, streptococcal lipoproteins together with H_2_O_2_ appear to be major factors underlying damage to human PDL cells caused by streptococcal species. Furthermore, macrophages infected by *E. faecalis* produce 4-hydroxynonenal (4-HNE; Wang et al., 2012b) likely *via* lipid oxidation. 4-HNE, on the other hand, is known to induce apoptotic cell death (Dalleau et al., 2013). Therefore, it is seemingly necessary to determine whether indirect effects *via* macrophages can affect oral streptococci-induced apoptosis.

In conclusion, we demonstrate that *S. gordonii* induces apoptosis *via* H_2_O_2_ production in PDL cells. *S. gordonii* lipoproteins are involved in the death of human PDL cells. *S. gordonii* is an opportunistic bacterium, commonly found in apical lesions of patients with apical periodontitis. Therefore, apoptotic cell death of PDL cells due to tissue damage caused by *S. gordonii* could influence the development of apical periodontitis.

## Data Availability Statement

The original contributions presented in the study are included in the article/Supplementary Material, further inquiries can be directed to the corresponding author.

## Ethics Statement

All experiments using healthy human PDL cells were approved by the Institutional Review Board at Seoul National University. The patients/participants provided their written informed consent to participate in this study.

## Author Contributions

SHH conceived the study. SHH and O-JP designed the experiments. O-JP, ARK, YJS, JI, HJJ, KBA, and SHH performed the experiments and interpreted the data. HSS and C-HY provided critical comments. All authors contributed to the article and approved the submitted version.

## Funding

This work was supported by grants from the National Research Foundation of Korea, which is funded by the Korean government (NRF-2019R1A2C2007041, NRF-2018R1A5A2024418, and NRF-2019R1I1A1A01060952).

## Conflict of Interest

The authors declare that the research was conducted in the absence of any commercial or financial relationships that could be construed as a potential conflict of interest.

## Publisher’s Note

All claims expressed in this article are solely those of the authors and do not necessarily represent those of their affiliated organizations, or those of the publisher, the editors and the reviewers. Any product that may be evaluated in this article, or claim that may be made by its manufacturer, is not guaranteed or endorsed by the publisher.
