# Supplementary material for: Induction of Apoptotic Cell Death by Oral Streptococci in Human Periodontal Ligament Cells
Source: Front Microbiol. 2021 Oct 14;12:738047. doi: 10.3389/fmicb.2021.738047 (PMC8551966; doi:10.3389/fmicb.2021.738047)
Supplement: Supplementary file 1 [file Presentation_1.pdf]

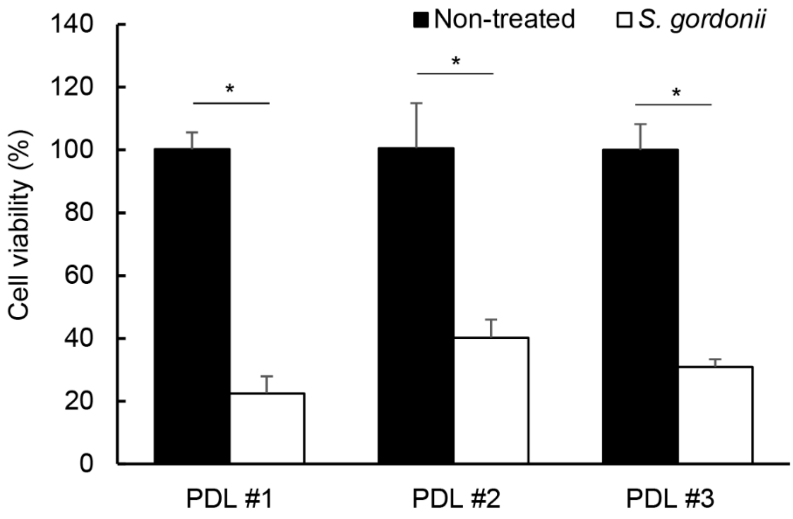

Supplementary Fig 1 *S. gordonii* efficiently induced cell death in all PDL cells. *S. gordonii* was cultured to mid-log phase in THY media at 37ºC. Then, PDL cells were treated with *S. gordonii* at MOI 1:1000 for 3 h. Trypan blue assay was used to determine the number of viable PDL cells. *$P < 0.05$.
